# Mechanotransduction through T cell receptors: consensus, controversies and future outlooks

**DOI:** 10.1038/s12276-026-01639-w

**Published:** 2026-02-05

**Authors:** Stefano Travaglino, Yelim Jeon, Yihyung Kim, Cheng Zhu, Hyun-Kyu Choi

**Affiliations:** 1 Wallace H. Coulter Department of Biomedical Engineering, Georgia Institute of Technology and Emory University, Atlanta, GA USA; 2 Parker H. Petit Institute for Bioengineering and Biosciences, Georgia Institute of Technology, Atlanta, GA USA; 3 Department of Biochemistry, College of Life Science and Biotechnology, Yonsei University, Seoul, South Korea; 4 George W. Woodruff School of Mechanical Engineering, Georgia Institute of Technology, Atlanta, GA USA

**Keywords:** Signal transduction, T-cell receptor

## Abstract

Immune cells rely on surface immunoreceptors to sense their environment. While the downstream signaling pathways of many immunoreceptors are well characterized, the initial molecular events that trigger signaling upon ligand engagement remain incompletely understood. Here, in this Review, we outline our current understanding of this immunoreceptor signal initiation problem, using the T cell antigen receptor (TCR) as a prototype. We synthesize decades of research on the TCR’s unique functional requirements and explore how these properties constrain potential triggering mechanisms. We evaluate prominent models of TCR signal initiation and highlight their respective strengths, limitations, complementary aspects and areas of ongoing debate. A central focus is the role of mechanical force in TCR triggering and antigen recognition for which we consider evidence for TCR–pMHC catch bonds, the capacity of T cells to generate endogenous forces and how these might modulate receptor–ligand kinetics and conformational changes to enhance antigen discrimination beyond classical kinetic proofreading models. By comparing TCR triggering with that of other immunoreceptors such as B cell receptors and Fc receptors, we discuss both shared principles and receptor-specific differences. This Review aims to consolidate current knowledge, reconcile conflicting findings and identify critical unanswered questions, in hopes of charting a path toward understanding how immunoreceptors convert ligand binding into cellular responses.

## T cell receptor mechanics enhance antigen recognition insights

The immune system protects the body using various cells that communicate through receptors. This Review explores how T cell receptors (TCRs) initiate signaling, focusing on their role as mechanosensors. The authors review current knowledge and controversies about TCR signaling. TCRs are crucial for recognizing antigens and activating T cells. The study examines different models explaining how TCRs initiate signaling. These include the aggregation model, conformational change model, kinetic segregation model and mechanosensor model. Research highlights that TCRs can detect mechanical forces, which may help them distinguish between different antigens. This ability could enhance the immune response’s sensitivity and specificity. The authors suggest that understanding these mechanisms could improve therapies targeting immune responses. Future research may focus on integrating these models to fully understand TCR signaling and its implications for immune system function and potential medical applications.

This summary was initially drafted using artificial intelligence, then revised and fact-checked by the author.

## Introduction

The human body is protected by the immune system relying on the diverse yet complementary roles of immune cells. Neutrophils, monocytes, eosinophils, basophils and natural killer cells participate in innate immunity, and T and B lymphocytes underpin adaptive immunity, while dendritic cells link the innate and adaptive immune systems^1–3^. Immune cells rely on surface receptors, including ion channel-linked receptors, G-protein-coupled receptors (GPCRs), adhesion receptors and immunoreceptors, to communicate with other cells and with their environment, thus enabling them to respond to external cues^4–7^. In contrast to ion channels and GPCRs, whose signaling is usually triggered by binding of soluble ligands (with a few exceptions such as Piezos^8,9^ and mechanosensitive GPCRs^10–12^), adhesion receptors and immunoreceptors signal upon binding to immobilized ligands on other cells or on the extracellular matrix^13–16^. It has been increasingly recognized that adhesion receptors and immunoreceptors play crucial roles in sensing, interpreting and responding to not only chemical but also physical cues rich in the environment of immune cells^17–22^. Compared with many ion channels and GPCRs, whose mechanisms of signal initiation are better understood, how ligand binding initiates signaling in most adhesion receptors and immunoreceptors of immune cells remains poorly understood.

In this Review, we examine the current understanding of how immunoreceptors initiate signaling, using the T cell receptor (TCR) as a prototype to illustrate key issues involved in what we refer to as the immunoreceptor signal initiation problem. We summarize the functional requirements, biochemical and biophysical properties, and conceptual and mathematical models of TCR triggering, signaling and function. In particular, we attempt to connect these requirements and properties to the potential role of the TCR (and, by extension, other immunoreceptors) that may function as mechanosensors. We aim to review the core issues, identify consensus and controversy, and suggest future studies needed to enable a solution to this problem. To help understand discrepant results and opposing views under debate, we discuss how different technologies, methodologies and sampling conditions used by different groups may influence their experimental findings. We also examine the underlying assumptions and interpretive issues that have shaped different schools of thought in the field. We begin by comparing the similarities and differences between immunoreceptors to identify the unique features of the TCR. We next summarize the main models of signal initiation by the TCR (and, to a lesser extent, other immunoreceptors). We then focus on the mechanosensor model of TCR activation (and, again, other immunoreceptors to a lesser extent), discussing whether immune cells actively generate endogenous forces through their immunoreceptors, exploring whether such forces can modulate receptor–ligand dissociation kinetics to elicit catch versus slip bond behaviors, and finally considering how these mechanical aspects influence immune cell signaling and functional outcomes. Through this mechanobiological lens, we aim to clarify which aspects of current models of immunoreceptor mechanotransduction are supported by definitive versus circumstantial evidence, which remain speculative, and how future work might reconcile existing controversies.

## The TCR and its unique features

**Fig. 1:**
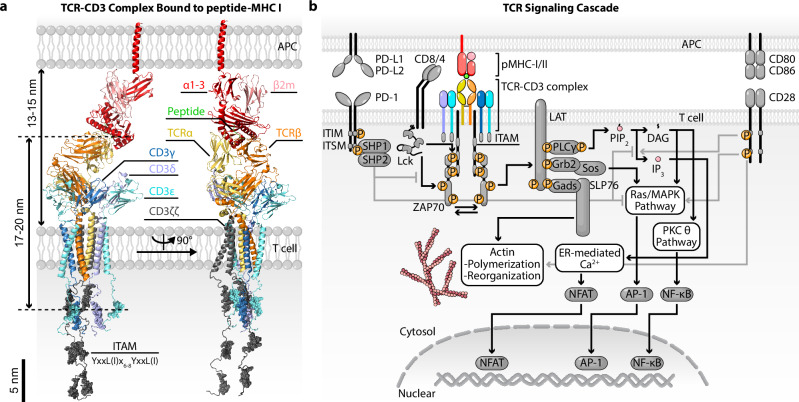
TCR structure and signaling pathway. **a** Structural illustration of the TCR–CD3 complex bound to a peptide–MHC class I (pMHC-I) molecule (modified from PDB: 8J8I and 7PHR). ITAM-associated residues are shown as spheres. Various globular domains are indicated and color-coded. Approximate molecular dimensions derived from PDB structures are indicated on the left. **b** Schematic of the TCR signaling pathways. Activating and inhibitory molecular circuits are indicated by black and gray arrows, respectively. ‘P’ denotes phosphorylation. Detailed signaling crosstalk is not shown. LCK, lymphocyte-specific protein tyrosine kinase; PIP_2_, phosphatidylinositol 4,5-bisphosphate; MAPK, mitogen-activated protein kinase; PKCθ, protein kinase Cθ; ER, endoplasmic reticulum ITI(S)M, immunoreceptor tyrosine-based inhibition(switch) motif); SHP 1/2, Src homology 1 or 2 domain-containing protein tyrosine phosphatase.

The TCR is arguably the most important immunoreceptor of T-cell-mediated adaptive immunity because its interaction with peptides presented by the major histocompatibility complex (pMHC) molecule allows antigen recognition by the T cell, leading to its activation, proliferation, differentiation and effector functions^23–26^. Structurally, the TCR is composed of two polymorphic ligand-binding chains (αβ or γδ) that associate noncovalently with three invariant signaling dimers: CD3γε, CD3δε and CD3ζζ (Fig. 1a). These signaling subunits contain immunoreceptor tyrosine-based activation motifs (ITAMs), which are phosphorylated upon ligand engagement to initiate downstream signaling cascades^27,28^. Specifically, the Src family kinase Lck, either free or membrane-anchored^29,30^ or associated with the cytoplasmic tails of the CD4 or CD8 coreceptors, phosphorylates tyrosine residues within the ITAMs of the CD3 subunits of the TCR complex^31,32^ (Fig. 1b). These phosphorylated ITAMs serve as docking sites for the tandem SH2 domains of the Syk family kinase ZAP-70 (zeta-chain-related protein kinase 70)^33^. Subsequent phosphorylation and activation of ZAP-70 by Lck enable ZAP-70 to phosphorylate the transmembrane (TM) adaptor protein LAT (linker for activation of T cells)^34^, which acts as a scaffold for assembling a multiprotein signaling complex or signalsome^35^. This complex includes molecules such as SLP76 (SH2 domain containing leukocyte protein of 76 kDa), Grb2 (growth factor receptor-bound protein 2), Sos (the guanine nucleotide exchange factor son of sevenless), Gads (Grb2-related adaptor downstream of Shc) and phospholipase Cγ (PLCγ)-1, leading to the activation of downstream signaling pathways including calcium mobilization (through inositol 1,4,5-trisphosphate (IP_3_)), protein kinase C (through diacylglycerol (DAG)), Ras/MAP kinase cascades (through Sos and DAG), actin reorganization (through SLP76) and finally activation of transcription factors such as nuclear factor of activated T cell (NFAT), nuclear factor-κB (NF-κB) and activating protein-1 (AP-1)^26^ (Fig. 1b). Amplification of these cascades is mediated by signaling of costimulatory receptors such as CD28^36,37^. Negative regulation of these processes is mediated by phosphatases such as SHP-1 and SHP-2, which are activated by inhibitory receptors such as programmed cell death protein 1 (PD-1) and dephosphorylate key signaling intermediates to modulate the intensity and duration of T cell activation^38^. Overall, these signaling events have been elucidated through extensive biochemical and genetic studies, establishing a foundational understanding of TCR-mediated signal transduction. Yet, this body of knowledge explains what happens only after TCR triggering, not how TCR triggering is initiated.

Several features of the TCR have been recognized as requirements for its function from four decades of extensive studies since its identification^39–58^. The most well-recognized three are: (1) Sensitivity—the TCR is exquisitely sensitive as T cells can detect a very low number of cognate pMHCs from a background of a large number of self pMHCs^40–42^; (2) specificity—such detection is highly specific as a single residue difference in the peptide can result in very different T cell response^42–47^; (3) discrimination—the TCR is able to discriminate self from nonself^41,42,45,48,49^. Discrimination is related to, but not identical to, specificity^50^. To discriminate two pMHCs, the TCR has to deliver two signals capable of generating two distinct T cell responses correctly, which requires the two signals to be accurately dissimilar. Specificity has a different and somewhat less stringent requirement: the TCR has to deliver an above-threshold signal to initiate a true response for one peptide but below-threshold signals for other peptides to avoid false-positive responses. In addition, three less mentioned features of the TCR have been observed: (4) speed—the T cell must make sufficiently rapid decisions based on the information received from the TCR–pMHC interaction for it to efficiently patrol the body to provide surveillance for pathogens and cancerous cells^51–53,58^; (5) dependency—TCR arguably has the greatest dependency compared with other immunoreceptors, as nearly all T cell functions depend on antigen stimulation through the TCR^55–57^; (6) commonality—features 1–5 are not limited to one or a few TCR sequences; rather, all TCR clones in an individual’s TCR repertoire share these features^54^. Some of the above features are unique to the TCR, while others are shared by other immunoreceptors. For example, both the TCR and B cell receptor (BCR) repertoires, estimated at 10⁵–10⁷ clonotypes, are generated through a shared mechanism of V(D)J recombination involving variable (V), diversity (D) and joining (J) gene segments^59,60^. Specificity, dependency and commonality are shared features of both antigen receptors. However, unlike TCRs, which recognize antigenic peptide fragments of proteins after their processing and presentation by MHC, BCRs directly bind to native or unprocessed antigens, enabling the recognition of a wide array of molecular structures, including proteins, polysaccharides and lipids on pathogens or Antigen-Presenting Cells (APCs). As another example, unlike TCR and BCR whose broad repertoires endow them the capability of recognizing any possible pathogenic and cancerous antigens, Fc receptors (FcRs) are germline-encoded and exhibit only limited polymorphism, with all humans expressing a restricted and conserved set of FcγRs, FcεRs, FcμRs, FcδRs and FcαRs to bind the constant Fc regions of antibodies (soluble forms of BCRs) of the immunoglobulin (Ig) G, E, M, D and A isotypes, respectively^61^. Using the variable Fab regions to recognize broad arrays of antigens, FcRs mediate conserved antibody-dependent effector cell functions upon binding to the constant Fc regions^61^.

Interestingly, despite their differences in antigen recognition mode, TCR, BCR and FcRs have all been observed to form a similar intimate cell–cell contact structure called immunological synapse (IS)^62–66^. In addition to the TCR, BCR or FcRs, different types of IS also involve other shared immunoreceptors, including costimulatory molecules, such as CD28 binding to CD80 and CD86, co-inhibitory molecules such as PD-1 binding to PD-L1 and PD-L2, adhesion molecules, such as lymphocyte function associated antigen-1 (LFA-1) binding to intercellular adhesion molecule-1 (ICAM-1), VLA-4 binding to VCAM-1, and CD2 binding to CD58, implying similar cross-intercellular junctional structures for stabilizing the IS organization and amplifying signaling^63,67,68^. The signal transduction of these immunoreceptors share many molecular components and pathways both within and beyond the IS, with their functions relying on membrane reorganization, cytoskeletal remodeling and spatial receptor clustering^69,70^. For example, upon antigen binding of TCRs, T cells rapidly remodel their actin cytoskeleton, initiating polymerization at the leading edge and generating retrograde flow that directs engaged receptors toward the synapse center^71–73^. Myosin-II-mediated contractions contribute additional force that shapes membrane curvature and sustains close-contact zones^74–77^. These cytoskeletal processes facilitate not only receptor clustering and signaling but also the physical exclusion of large phosphatases such as CD45, thereby shifting the local kinase–phosphatase balance in favor of activation^78,79^. Furthermore, the TCR–CD3 complex is driven to move laterally on the T cell surface to organize into macromolecular assemblies with other molecules at and beneath the membrane^80–82^. Whereas whether the ligand-binding TCRαβ chain is connected directly or indirectly via adaptor proteins to the actin cytoskeleton remains unclear, CD3 signaling chains may be indirectly associated with the cortical actin network via ERM proteins such as ezrin, which help to organize the structural environment necessary for IS formation^83,84^, suggesting the presence of mechanical force between TCRαβ and CD3s. A similar ambiguity surrounds the BCR and FcRs, both of which display cytoskeleton-sensitive signaling and localization but no confirmed direct or indirect mechanical linkages^63,85,86^.

Despite the many shared features and properties with other immunoreceptors, what sets the TCR apart is not only its ligand restriction and signaling architecture but also the functional imperatives derived from its role in immune surveillance. To discriminate nonself from self with exquisite sensitivity, specificity and speed across the immense and individually distinct repertoire of TCRs, the TCR must achieve a high level of molecular accuracy not required by other immunoreceptors.

## Models of Tcr triggering and antigen discrimination

### Tcr triggering models

**Fig. 2:**
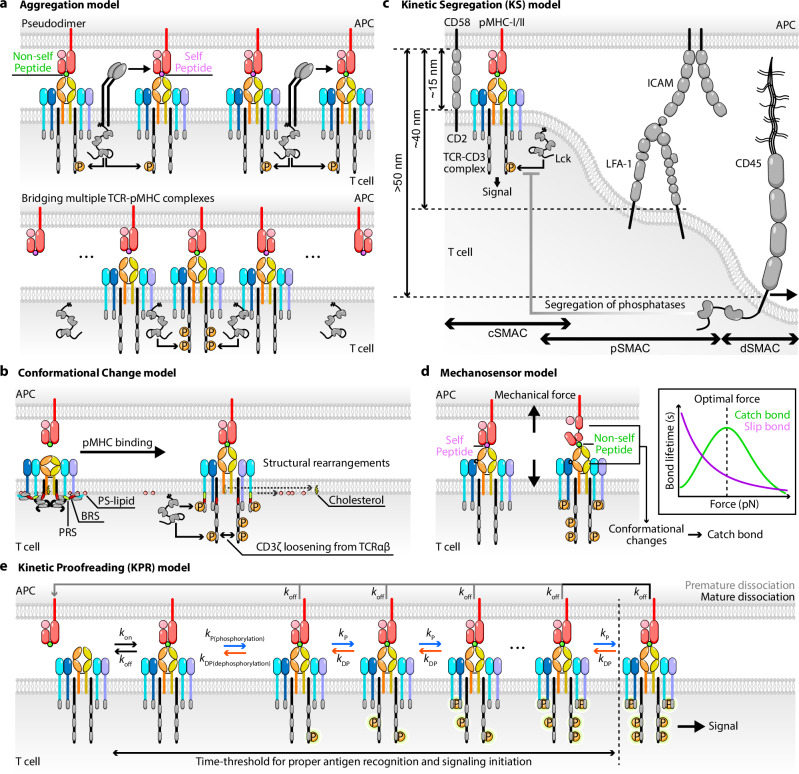
Models of TCR triggering and T cell antigen recognition. **a** Aggregation model. TCR signaling is initiated by pseudodimer formation (top left) or coreceptor heterodimerization (top right) of TCR–pMHC complexes, leading to TCR microcluster formation and promoting ITAM phosphorylation on CD3 cytoplasmic tails. **b** Conformational change model. TCR–pMHC binding induces structural rearrangements within the TCR–CD3 complex, facilitating ITAM phosphorylation on CD3 tails. **c** KS model. Spatial exclusion of large phosphatases (for example, CD45) from the TCR–pMHC microcluster enables Lck-mediated phosphorylation and signal initiation at the close-contact interface. **d** Mechanosensor model. Mechanical forces exerted on TCR via engaged ligands distinguish between self and nonself pMHCs, promoting conformational changes and eliciting catch bond with nonself pMHCs and slip bond with self-pMHCs. **e** KPR model. Sequential signaling steps require sustained TCR–pMHC engagement. Only ligands with dwell times exceeding a threshold can complete the proofreading cascade and initiate full T cell activation. ${k}_{\mathrm{on},\mathrm{off}}$, on/off rates of TCR–pMHC binding; ${k}_{{\rm{P}},\mathrm{DP}}$, phosphorylation (P) or dephosphorylation (DP) rates of CD3s.

It is generally believed that the ability of TCR to exhibit the aforementioned features lies, at least partly, in the mechanism of TCR signal initiation, or TCR triggering. Although the TCR triggering mechanism is not fully understood, it is constrained by these TCR features, that is, requirements for the TCR triggering mechanism. The mechanism of signal initiation has to explain how the pMHC binding event is transmitted from the ligand binding site at the N-terminal end of the TCRαβ over 17–20 nm of space spanning from the middle of the intercellular gap across the T cell membrane to the CD3 cytoplasmic tails to trigger ITAM phosphorylation events, which is too far a distance for chemical reaction to bridge (Fig. 1). This is a unresolved problem not only for the TCR but also for all other immunoreceptors^87^. In the following, we discuss four mechanistic models of TCR signaling initiation^88–90^ (Fig. 2a–d).

#### Aggregation model

This model suggests that binding multimeric ligands brings immunoreceptor into clusters, increasing the local concentrations of pertinent molecules and driving chemical reactions forward by virtue of mass action to initiate signaling. It addresses three requirements for signal initiation: the availability, close proximity and sufficient concentration of molecular players for the triggering event. It is therefore commonly proposed for many receptors, including TCR^91^, BCR^92^ and FcRs^93^. Perhaps the best-understood example of this mechanism is the epidermal growth factor (EGF) receptor, whose signal initiation model proposes that EGF binding causes receptor dimerization to bring the kinase domains of the two receptors into close proximity, allowing one kinase domain to phosphorylate the other, that is, trans-autophosphorylation^94,95^. Notwithstanding the lack of structural evidence, a coreceptor-mediated heterodimerization, or pseudodimer, model has been proposed for TCR triggering^96,97^ (Fig. 2a). Supporting evidence for this model comes from multiple lines of experimental evidence^98,99^. For instance, TCR triggering has been observed under low agonist pMHC density when microclusters form at the IS, implying that local receptor density may compensate for limited ligand availability. Notably, soluble pMHC tetramer^100^ and Ig-dimer^101,102^, but not pMHC monomer^103,104^, trigger T cell signaling, despite all three pMHC forms can bind TCR (and their different binding propensities can be compensated by concentrations), emphasizing the importance of membrane and spatial constraints^98^. Similar to the EGF receptor model, the pseudodimer model proposes that coreceptor-associated Lck may facilitate signal initiation by bridging together multiple TCR–pMHC complexes^96,97^. Furthermore, a high background of self-pMHC molecules (up to 50–100%) can serve as co-agonists to enhance T cell recognition of agonist pMHC, probably by increasing the probability of microcluster formation and stabilizing early signaling complexes^105,106^. The idea of TCR clustering is supported by observations that TCRs exist as pre-organized nanoclusters before antigen engagement^107–110^. These nanoclusters enable cooperative ligand binding, where initial TCR–pMHC interactions promote subsequent binding within the same cluster. Despite these experimental supports, the aggregation model suffers several limitations. More fundamentally, it does not account for the exquisite sensitivity of the TCR, namely, even a single agonist pMHC bound to TCR can trigger full T cell activation^104,111,112^, negating the necessity of receptor clustering. In addition, the aggregation model lacks mechanisms to explain TCR specificity and discrimination between pMHC ligands, especially when the phenomenon of co-agonism is considered.

#### Conformational change model

This model suggests that pMHC binding results in either/both direct and/or allosteric changes of the TCR–CD3 complex, transmitted from the ligand-binding loops in the TCRαβ (or TCRγδ) subunits to the cytoplasmic tails of the CD3 subunits^90,113–117^ (Fig. 2b). Before the first publication of the cryo-electron microscopy (cryo-EM) structure of the complete TCR–CD3 complex^118^, early studies demonstrated that ligand binding to the TCR induces a conformational change in the CD3ε subunit, resulting in the exposure of a proline-rich sequence (PRS) recognized by adaptor proteins such as Nck or by the conformation-sensitive antibody APA1/1^119–121^. Subsequent studies extended this concept to additional subunits, including CD3ζ, indicating that multiple components of the TCR–CD3 complex undergo structural rearrangements upon engagement^122^. Supporting this, fluorescence-based studies have shown that, in the resting state, the ITAMs of CD3ε and CD3ζ are embedded within the plasma membrane, thereby limiting access to Src family kinases^123,124^. A natural next question is how signaling is initiated through conformational unmasking (that is, how the cytoplasmic tails of CD3ε and CD3ζ detach from their membrane anchorage), which is mediated by electrostatic interactions between basic-residue-rich sequences (BRS) and phosphatidylserine (PS), to make their ITAMs available to kinases for phosphorylation^123,124^. An early study suggested that, upon pMHC binding, the inner leaflet charge becomes less negative due to the local depletion of PS from TCR microclusters^125^. A later study proposed that this connection is disrupted by Ca^2+^ ions, which modulate the lipid charge properties^126^. More recent work reconciles these findings by showing that, upon TCR stimulation, the Ca^2+^-dependent lipid scramblase TMEM16F redistributes PS from the inner to the outer leaflet of the plasma membrane^127^. This mechanism incorporates elements of both earlier hypotheses while clarifying that Ca²⁺ does not directly neutralize PS charge^127^. Regardless of the exact mechanism, this PS redistribution and CD3 tail detachment appears to involve bystander TCRs and amplify signaling but does not yet satisfactorily explain how the CD3 tails detach upon pMHC binding. While this class of models accounts for the shielding of CD3ε and CD3ζ in the resting TCR and their exposure following ligand binding, it fails to explain the resting-state masking of the CD3δ and CD3γ tails, which do not bind the inner leaflet of the membrane and would therefore be expected to be exposed in the resting state. Moreover, shielding of the CD3 tails was observed even in detergent-solubilized TCR, where Lck could phosphorylate CD3 ITAMs in the presence, but not in the absence, of ligand binding to TCR, casting doubts on the necessity of the interactions of the CD3 tails with the plasma membrane^128^. An alternative model has been proposed to address some of these issues by positing that, in the resting TCR, the CD3 tails shield one another, resulting in auto-inhibition of the signaling motifs. In this permissive geometry model of TCR activation, a dimeric pMHC binding two pre-multimerized TCRs (or bringing two monomeric TCRs close to each other) would cause a rotation of the two TCRαβ ectodomains with respect to one another and a subsequent scissor-like motion of the CD3 tails, resulting in exposure of the ITAMs for phosphorylation^129^.

While the role of the TCR TM domain was traditionally thought to be limited to forming the quaternary TCR–CD3 complex and mediating solvation in the plasma membrane, more recently the TM domain—and, to a lesser extent, the connecting peptides (CPs)—has gained attention as a key regulator of TCR conformation and allostery^115,117,128,130^. Importantly, the interactions of the TM with lipids, particularly with cholesterol, appear to have a key role in allosteric switch between resting and active TCR conformations^117,128^. Namely, cholesterol was found to interact with the TCRβ TM helix and keep the TCR in a resting, inactive conformation (TCR_r_). Cholesterol, when bound to TCR, stabilizes the TCR_r_ conformation, preventing ITAM phosphorylation and TCR signaling in the absence of pMHC. Spontaneous unbinding of cholesterol from TCRβ switches TCR to a primed conformation (TCR_p_), which allows ITAM phosphorylation and TCR signaling when ligand is engaged. Conversely, binding to pMHC stabilizes TCR in the TCR_p_ conformation. Recently, a full cryo-EM structure of the TCR, including the TM domain, resolved two cholesterol molecules within the core TM tunnel, interacting with TCRβ, CD3γ and CD3ζ^130^. Importantly, cholesterol appears to reduce TM motility and, by ‘latching’ on CD3ζ, prevent CD3ζ from assuming an active conformation. Moreover, mutagenesis of key residues in the TCRβ TM found to interact with cholesterol leads to increased TCR signaling both without (auto-activation) and with ligand stimulation compared with the wild-type structure^130^. In addition, it was recently reported that even a single amino acid substitution in either the CD3ε ectodomain or the CD3ζ/TCRβ TM domain can respectively prevent or facilitate the allosteric switch that triggers TCR signaling^114,131,132^. Two mutations to the extracellular domain of CD3ε in the CP region were found to prevent the transmission of conformational changes upon ligand binding and prevent the switch from TCR_r_ to TCR_p_^128,131,132^. Conversely, mutations to the TM that loosened the cohesion of TCRαβ with CD3ζ were found to be gain of function and to enhance TCR signaling^114^. Similar to mutations that loosened cohesion between TCRαβ and CD3ζ, engagement of TCR (either membrane bound or solubilized) by soluble monovalent pMHC leads to quaternary structure relaxation^107^. Taking together, these findings provide compelling evidence that ligand binding can either induce long-range changes in the conformation of TCR or at least stabilize active, signaling-competent TCR conformations. Building on the static cryo-EM structure^118^, molecular dynamics (MD) simulations have also provided insights into the conformational and allosteric changes within the TCR–CD3 complex upon pMHC engagement, revealing that ligand binding induced TCR quaternary structure relaxation such as loosening of CD3ζ from TCRαβ, which results in enhancement of CD3ζ phosphorylation^114,115^. Again, interactions between CD3 domains and membrane structures have been shown to be important for allosteric switching at the atomic level^106^. Thus, conformational change models outline a signal transmission process from the binding event at the membrane-distal end of the ectodomain of the TCR to the physical changes in its TM and cytoplasmic domains that involve interaction with and regulation by lipids in the membrane. However, controversies still exist because a fully assembled TCR–pMHC structure reconstituted in detergent showed no conformational change compared with the unliganded TCR–CD3 complex^133^. By contrast, using nanodisc-based reconstitution, new data suggest that the unliganded TCR–CD3 adopts a more closed and compact conformation as its resting state, and that liganded structure displays an open and extended conformation. The authors suggest that ectodomain opening is required for full T cell activation^134^. These current versions of conformational change model based on cryo-EM and MD simulations remain incomplete as they have yet to fully account for the unique features of TCR signaling. For instance, they do not address how cognate antigens are preferentially recognized by the TCR (sensitivity), nor do they explain how partial agonists convey distinct signaling outcomes through the CD3 dynamics compared with agonists (discrimination). While the general concepts of the conformational change model may be extensible, many of the specific details may not be generalizable to other immunoreceptors. Nonetheless, conformational change models can be synergistically incorporated with other models such as the aggregation model. For example, it has been shown that multivalent pMHC ligands induce a transient enhancement of TCR–pMHC binding within a 4- to 8-min window, correlating with the adoption of a conformational active state of clustered TCRs, as measured by monoclonal antibody APA1/1 binding to the CD3ε activation epitope^107^. Importantly, cholesterol depletion—which disrupts nanoclusters—abolishes this cooperativity, highlighting that physical clustering is required for signal amplification^107^. TCR nanoclusters transition cooperatively into an ‘active’ conformation upon tetrameric pMHC binding. This conformational change propagates across neighboring TCRs within a cluster, even to unliganded receptors, suggesting lateral allosteric communication. Hence, the conformational change of TCR–CD3 is not merely a molecular event within a single TCR–pMHC complex but a coordinated process at the level of nanocluster architecture that promotes aggregation, offering a mechanistic bridge between clustering and conformational triggering^107^.

#### Kinetic segregation (KS) model

This model can be illustrated using the structural organization of the established IS. The IS structure reflects the dynamic rearrangement of immunoreceptors, adhesion molecules and cytoskeleton and is organized into supramolecular activation clusters (SMACs)-central (cSMAC), peripheral (pSMAC) and distal (dSMAC). TCR–pMHC bonds, which have a dimension of 13–15 nm, are concentrated in the cSMAC together with CD2–CD58 bonds that have a similar dimension and act as adhesion pairs to form ‘close contacts’^135,136^. These close contacts help maintain an optimal membrane distance and enhance the scanning activity of T cell microvilli. Having a dimension of ~40 nm, bonds between extended LFA-1 (α_L_β_2_) on T cells and ICAMs on APC move from the center to the pSMAC during IS formation, which is crucial to adhesion stabilization of the IS^135,137,138^. By contrast, bulky glycocalyx molecules such as CD45 (an orphan receptor with no known ligand, a ~50-nm ectodomain and a cytoplasmic domain containing tyrosine phosphatase catalytic activity) are pushed further outside to the dSMAC^78,135,139^ (Fig. 2c). However, initial TCR signaling is triggered in TCR-containing nanoclusters in the periphery of the T cell–APC contact before full development of IS bull’s-eye pattern, and the cSMAC is where TCR is downregulated to terminate signaling^140,141^. Extending the observation from the established IS to the situation where even a single TCR bond with monomeric pMHC can trigger TCR signaling^142^, the KS model postulates that, before the formation of close contacts between T cell and APC, the phosphorylation of ITAMs by Lck is balanced with dephosphorylation by CD45 phosphatases. After the formation of a TCR–pMHC bond, CD45 is excluded from the vicinity of the TCR, thereby tilting the balance between kinases and phosphatases toward favoring the former and resulting in ITAM phosphorylation^78,79,141,143–147^ (Fig. 2c). Supporting evidence has been generated by examining how TCR triggering is altered by changing the exclusion of key signaling components. For example, changing the ectodomain dimension of CD45 has been shown to influence both Lck activity and TCR signaling^146^. A CD45 chimera incorporating the large ectodomain of CD43 can restore TCR signaling in CD45-deficient T cells, whereas chimeras containing smaller ectodomains from other phosphatases fail to do so. NFAT activation in response to stimulation by soluble anti-TCR antibodies is inhibited by expressing CD148 (another phosphatase with sizable ectodomain) on T cells, but not when stimulation is provided by immobilized pMHC (expressed on APCs) or anti-TCR antibodies (coated on glass)^147^. Increasing the size of the pMHC ectodomain, which decreases the size difference of CD45 relative to the TCR–pMHC bond and is hence predicted to decrease the driving force of CD45 exclusion, significantly reduces TCR triggering without affecting ligand binding^143^. Notably, this diminished activation can be rescued by enhancing the affinity of the interaction, suggesting that propensity of this interaction plays a critical role in TCR signal initiation^148^. Moreover, reconstitution-based assays have shown that the binding strength between TCR and pMHC can produce a repulsive effect on surface proteins bearing bulky or unengaged ectodomains, even when intracellular signaling cascades are not engaged^149^. This observation indicates that the accumulated binding energy of cross-membrane bonds can drive the spatial segregation of receptors of different sizes regardless of whether they form crossbridges with counter-receptors, and implies that coreceptors and cytoskeletal linkers may be dispensable for TCR triggering upon pMHC binding across cell membranes, although they appear important for sharpening antigen discrimination and ensuring responsiveness at minimal stimulus levels. Nevertheless, some evidence suggests that even before the formation of a mature IS, CD45 is pre-excluded from the tips of T cell microvilli, where TCRs are concentrated, and instead localizes along the shafts of the microvilli, creating a phosphatase-depleted zone at the tip that permits rapid TCR triggering^150^. Furthermore, studies investigating three-dimensional (3D) structures of T cell membrane protrusions including microvilli, and invadosome-like protrusion have increasingly emphasized the importance of ultrastructural configurations for proper early TCR signaling, relation between microcluster and microvilli, and KS in the large size of protrusion^151^.

Conceptually, the KS model is based on the physical reasoning of size exclusion of the receptor ectodomains, which in turn changes the relative concentrations of kinases versus phosphatases to initiate signaling. As such, it seems applicable to other immunoreceptor triggering^78^. Indeed, the proposers of the KS model have extended it to be a general mechanism for various receptors^152–155^. For example, mitogenic ‘super-agonist’ antibodies, such as anti-CD28, bind to specific epitopes that create compact complexes, excluding large phosphatases such as CD45 and enabling receptor phosphorylation^152,153^. This model accounts for why antibody-induced signaling requires immobilization and suggests that native ligands such as B7-1/B7-2 may trigger CD28 in a KS-like manner. As most known membrane tyrosine phosphatases possess large extracellular domains^156^, whereas many immunoreceptor–ligand pairs are comparable in size to the TCR–pMHC complex, it is possible that TCR triggering is not unique but part of a broader class of KS-regulated cell–cell recognition systems. This concept has even been extended to other receptor classes, such as Notch, in which size-dependent segregation from the RIP microdomain shows notable parallels to the KS model of immune cell activation^155^.

#### Mechanosensor model

This model proposes that mechanical force applied on the TCR–pMHC bond triggers TCR signaling^90,91,104,157–161^ (Fig. 2d). It is supported by experiments that monitor intracellular calcium while concurrently exerting mechanical force on TCRs, showing that piconewton forces on single TCR–pMHC bonds can trigger T cell signaling^104,157,162–164^. Importantly, mechanical force modulates TCR–pMHC dissociation, generating catch bonds—where force strengthens the interaction and prolongs its lifetime—or slip bonds, where force weakens the interaction and shortens its lifetime^165^, depending on the biological activity of the ligand^42,104,158,162,166–170^. Intriguingly, parallel experiments comparing αβ versus γδ TCRs show that force elicits catch bond in the former, but not the latter, TCR in a manner that correspond to their functionality^171,172^. While these data demonstrate force can trigger and amplify TCR signaling, other data suggest that force may not be required for TCR triggering^157,162^, as soluble multimeric pMHC to TCR induces signaling^100^. Although it may not be a ‘professional’ mechanosensor like Piezo channels^8,9^, the TCR can still function as a mechanosensor because it is mechanosensitive and mechanoresponsive, even though it can also be triggered by ligand binding in the absence of force. In addition, the mechanosensor model helps to explain the sensitivity, specificity and discrimination of TCR recognition. However, how mechanical force is transmitted to the CD3 cytoplasmic domains where signaling is initiated and how such force-based mechanisms are reconciled with other models requires further consideration.

Each of the above models offers distinct strengths but also has limitations. No consensus has been reached regarding which model best accounts for all aspects of TCR triggering. However, these models are not mutually exclusive and can be integrated, as noted earlier with respect to the aggregation and conformational change models. As another example, tensile force must be induced on the TCR–pMHC bonds as a reaction to the compressive forces that squeeze CD45 out of the contact zone, providing a connection between the KS model and mechanosensor model. Perhaps the most complementary models are those of mechanosensor and conformational change as it is intuitive that force may deform molecular structures and induce conformational changes. Indeed, pulling on the TCR–pMHC bond has resulted in a sudden increase in molecular length, suggestive of conformational changes in the TCR, the pMHC, or both^158,166,167^. These observations are further supported by steered MD simulations, which show that force applied to TCR bonds with agonist pMHCs elicits catch bonds that enhance mechanical stability by inducing conformational rearrangements within the TCR–pMHC complex. By contrast, such rearrangements are not observed in TCR bonds with antagonist pMHCs, resulting in slip bonds that reduce mechanical stability^167,173^. To initiate signaling, allosteric changes induced by pMHC binding must propagate through the TCRαβ, across the membrane, and be relayed to the CD3 subunits to induce conformational changes^114,116^. Therefore, the pMHC-encoded information must be transmitted through the same molecular junction between TCRαβ and the CD3 subunits and, as such, should be incorporated into both the conformational change and mechanosensor models. One plausible integration of the mechanosensor model and conformational change model may be that mechanical forces propagate through the TCR–pMHC complex to induce structural rearrangements in TCRαβ and CD3 subunits, thereby stabilizing the putative active TCR conformation, or even facilitating exposure of ITAMs for phosphorylation by mechanical detachments of CD3ε and CD3ζ from the membrane.

### Models of T cell antigen discrimination

None of the TCR triggering models can adequately account for the TCR’s unique feature of antigen discrimination without including model elements other than the TCR itself. As a feature shared by all immunoreceptors, specificity requires only that the TCR bind pMHC in a binary fashion, that is, cognate versus noncognate. By comparison, accurate discrimination requires the TCR to decode the rich information contents encoded in a pMHC to induce nonbinary T cell responses, such as agonist, partial agonist, antagonist and anergic. While it remains unclear what specific differences the TCR senses when interacting with different pMHCs, the general approach has been to measure interaction characteristics and examine their correlation—or lack thereof—with the T cell responses they elicit. Many TCR–pMHC interaction characteristics have been investigated, including structural features of the contact interface, such as the docking angle between the two molecules^90,174,175^, mechanical properties, such as flexibility^176^, thermodynamic properties, such as Gibbs free energy^177^, interaction properties, such as binding affinity^178^, and, most commonly, kinetic rates in the absence and presence of force^165^. Notwithstanding outliers, many characteristics have indeed been found to display variable degrees of correlation with T cell signaling and function, suggesting that they contain, at least partly, the instruction that the TCR–pMHC interaction provides to the T cell to direct its response. For example, it seems reasonable to associate binding affinity with sensitivity, because the higher the affinity, the lower the pMHC density required for the TCR to form bonds with pMHC. This may be the case for affinity-maturated BCR whose equilibrium dissociation constant ${K}_{{\rm{D}}}$ (reciprocal affinity) is of the order of nanomolar^179^. However, TCR typically has a micromolar ${K}_{{\rm{D}}}$ and does not maturate^180^. As another example, the off-rate (or its reciprocal, the bond lifetime) has commonly been associated with antigen discrimination, based on the principle of kinetic proofreading (KPR), which posits that a minimum time is required for the TCR to complete the activation process^52,181,182^.

Existing models of T cell antigen recognition are based on, or modified from, the KPR model^50,183–186^ (Fig. 2e). The idea of KPR model is that recognition involves not only the TCR itself but also other intracellular signaling molecules that bind and enzymatically modify it, which is a process that requires time. It hypothesizes that pMHC binding results in a series of reversible chemical events (for example, binding and enzymatic modifications) that occur downstream of TCR triggering in the signaling pathway. To recognize the antigen, these biochemical events must proceed consecutively through an interaction network up to a decision point of no return. Yet, not only the initiation but also the maintenance of this reaction-kinetic process requires continuous engagement of the TCR with pMHC. Premature dissociation of the TCR–pMHC complex is assumed to terminate the serial reaction chain, resulting in failure of recognition and resetting the system back to its initial state to wait for the next TCR–pMHC binding event to restart the process again. The concepts of mature versus premature dissociation define a time threshold for proper antigen recognition and signaling initiation. Studies aiming to identify the biochemical events that may act as rate-limiting steps accounting for a substantial portion of the time within this threshold have found that multiple biochemical processes, such as coreceptor engagement coupled to Lck, full activation of ZAP-70 leading to LAT signalosome formation, and LAT phosphorylation, are potential candidates for this molecular timer^187–189^ (Fig. 1b).

In the initial KPR model, this time threshold is formulated using a minimum bond dwell time reciprocally related to the TCR–pMHC dissociation off-rates. Studies measuring the time threshold have indicated that inducing substantial T cell function via TCR stimulation with pMHC requires a minimum duration on the order of seconds^53,190–192^. This is consistent with many of the dwell times measured using surface plasmon resonance (SPR)^40,49,191^ but longer than most of the average bond lifetimes measured on the T cell surface using biomembrane force probe (BFP)^42,104,167^. Concurrent measurement of single-bond lifetime and calcium imaging on the same T cells suggested that continuous TCR–pMHC engagement beyond a threshold time is not required to trigger intracellular calcium flux; instead, the requirement may be that lifetimes from a sequence of intermittent bonds accumulate over a time window exceeding a threshold time^104^. This finding is consistent with the serial engagement model, which was originally proposed to overcome the sensitivity vs specificity trade-off^193^. The sensitivity feature allows the TCR to be triggered by a very low number of foreign peptides, suggesting a low triggering threshold. Yet, to maintain high specificity, the TCR must not be triggered by the overwhelming number of self-peptides, which also provide weak stimulation to the TCR. The serial engagement model suggests that the cognate pMHC has an optimal bond lifetime to allow it to dissociate from one TCR after its triggering, and to engage another TCR to trigger it consecutively, and so on and so forth^193–195^. This suggests a modification of the KPR concept, in which the series of reversible chemical events can only proceed downstream if sufficiently high concentrations of reactants (activated signaling molecules) accumulate to drive the reaction forward. These concentrations are built up and maintained by serial TCR–pMHC engagements, each generating a certain amount of reactants depending on its lifetime, until the system reaches the point of no return. If the reactant supply cannot meet the demand, the reactions cease. A modified KPR model has been proposed to include serial engagement due to rebinding^196^, which has the potential to account for the experimental findings^104^. Most recently, the serial bonds has been further extended to incorporate additional complexity by considering the binding kinetics between the TCR–coreceptor (CD8) complex and pMHCs^197,198^.

The mathematical formulation of KPR amplifies the off-rate difference in a highly nonlinear fashion, enabling the model prediction to relate very small, experimentally measured, differences in TCR–pMHC off-rates to very large difference in T cell function mediated by these TCR–pMHC interactions^50,186,199–201^. Yet, KPR as a time-based model is unable to explain all aspects of complex T cell responses. It fails to explain low-affinity tonic TCR signals, in which persistent, low-level interactions with self-antigens play a critical role in sustaining T cell viability, homeostasis and antigen responsiveness. Perhaps the most recognized difficulty of the KPR model is its reliance on deterministic off-rates, which is an ensemble averaged property. It has long been noticed that individual measurements of TCR–pMHC interactions, such as bond lifetime, are highly stochastic with broadly distributed values. Yet, the characteristics used to correlate with T cell responses are deterministic, as they are either measured in population assays or derived from statistics of many individual measurements; for example, the off-rate is calculated as the reciprocal of the average bond lifetime^104^. Although there are small differences in the statistically stable characteristic values of different TCR–pMHC pairs, large overlaps often exist in the distributions of their individual measurements^168^. This presents a conceptual challenge because, if TCR can be triggered by a single pMHC bond, it is unclear how the T cell can overcome the ‘noise’ in the random outcomes of individual interaction events to maintain fidelity to the distinctive functional responses. One possibility is that individual T cell responses mirror the outcomes of individual TCR–pMHC interactions, displaying the same stochastic behavior, and appear deterministic only in population assays that average over this stochasticity. It is also possible that an individual T cell response results from a series of individual interactions and the information delivered by each interaction is accumulated and integrated by the cell’s signaling network to reach a deterministic decision. A third possibility is that the TCR may respond only to rare extreme values, which can exhibit larger differences between measurement ensembles than the averages, or that only the so-called digital TCRs respond to the small number of cognate pMHC molecules^162^.

Interestingly, integrating the KPR model with the mechanosensor model helps overcome the above difficulty owing to an important aspect of the mechanosensor model, which is that the TCR forms catch-slip bonds with agonist pMHCs, but slip-only bonds with antagonist pMHCs^42,104,158,162,166–170^. Force prolongs the lifetimes of TCR bonds with agonist pMHCs that form catch bonds, and shortens the lifetimes of TCR bonds with non-agonist pMHCs that form slip bonds, essentially widening the lifetime differences between agonist and non-agonist pMHCs. Measured peak bond lifetimes of various catch-slip bonds formed between TCR and agonist pMHCs range from 1 to 10 s (refs. ^158,162^), enabling the TCR–CD3 complex to surpass the time threshold. Moreover, force enhances the cooperation between TCR and coreceptor for pMHC binding by significantly lengthening the lifetime of trimolecular bonds of TCR and coreceptor with agonist pMHC or even converting the bond type of TCR–pMHC bimolecular slip-only bonds to TCR–pMHC–coreceptor catch-slip bonds. This, in turn, greatly amplifies the differences in TCR engagement lifetimes between agonists and antagonists^159,168,202^. This peptide quality-dependent bond type switch between catch and slip behaviors provides a mechanism to ease the challenge of the KPR model to resolve subtle off-rate differences under zero-force conditions, as force can increase off-rate differences and thus enhance the discriminatory power of the TCR. However, this integrated KPR and mechanosensor model still cannot explain all experimental observations. For example, some signal-inducing pMHCs form catch-slip bonds with TCRs but display shorter lifetimes than nonstimulatory pMHCs that form slip-only bonds with the same TCRs, even within the optimal force range^158,170^. These observations raise important questions, such as what molecular mechanisms underlie the correlation between TCR–pMHC bond type and T cell signaling capacity, and what specific role the 10–20 pN force range plays in TCR mechanotransduction. One plausible explanation, supported by recent studies, is that mechanical tension induces conformational changes in either or both the TCR and the agonist pMHC, resulting in the rearrangement of molecular interactions at the binding interface and the formation of catch-slip bonds that favor TCR signaling initiation^158,165^. By contrast, mechanical tension may dissociate TCR and the antagonist pMHC bond without inducing conformational changes, resulting in the formation of slip-only bonds^158,165^.

### Comparison with signal initiatioin of other immunoreceptors

Structurally, BCRs are symmetrically similar to TCRs. Both lack intrinsic signaling motifs in their antigen-binding chains and rely on associated signaling subunits (CD3 for TCRs, CD79 for BCRs) containing ITAMs for signal transduction. Similarly, FcγRIIIA lacks intrinsic signaling motifs but associates with ITAM-containing cytoplasmic γ-γ or ζ-ζ dimeric chains. Meanwhile, FcgRIIA and FcgRIIB have ITAM and ITIM, respectively, in their cytoplasmic tails. In addition to signaling motifs, TCRs, BCRs and FcRs share common downstream signaling components. For example, Src family kinases (for example, Lck and Lyn), Syk family kinases (for example, ZAP-70 and Syk), and adaptor proteins (for example, LAT and SLP-76) lead to activations of transcription factors such as NF-κB and NFAT for inflammation, cell survival and immune responses. With the exception of the TCR, signal initiation by most other immunoreceptors is conceptually less complex. In the case of BCR, several mechanistic models have been proposed, including the cross-linking model (clustering-based), the dissociation-activation model (involving ITAM exposure), the force-dependent conformation-induced oligomerization model, and the antigen-footprint model, all of which bear conceptual parallels to corresponding models of TCR triggering^87^. FcRs have also similar triggering models including clustering, coligation and conformational change models^203^. In contrast to FcR activation, which may rely on relatively low ligand specificity, TCRs and BCRs are distinctly required to recognize ligands with minimal structural differences (such as a single amino acid substitution) that can lead to drastically different immunological outcomes. Thus, the mechanism of antigen recognition by the TCR (and BCR) must also explain its ability to discriminate, that is, how the information differentially encoded in the amino acid sequence of the peptide is transduced into differential phosphorylation patterns of the CD3 ITAMs and further decoded by other signaling molecules to direct distinct T cell downstream functions.

## Issues of the mechanosensor model

### Whether and how do catch versus slip bonds relate to binding affinity?

**Fig. 3:**
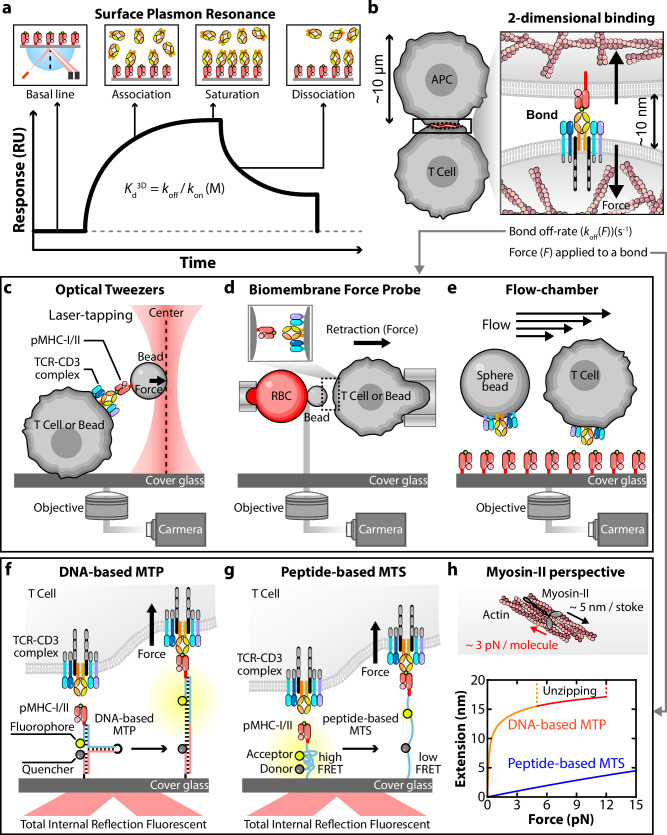
Biophysical techniques for measuring protein–protein interaction and endogenous force. **a** SPR. SPR measures 3D binding kinetics between purified TCR and pMHC in a cell-free system. The association and dissociation phases yield the on-rate (${k}_{\mathrm{on}}$) and off-rate (${k}_{\mathrm{off}}$), enabling calculation of the 3D equilibrium dissociation constant (${K}_{{\rm{d}}}^{3{\rm{D}}}$). **b** 2D interaction under cell–cell contacts. Physiological T cell–APC interfaces involve 2D interactions subjected to mechanical force ($F$). The bond dissociation rate (${k}_{\mathrm{off}}(F)$) reflects force-dependent unbinding behavior at the T cell–APC interface. **c**–**e** SM force spectroscopy techniques for measuring force-dependent off-rates of TCR–pMHC bonds. OTs (**c**); a laser trap manipulates beads coated with pMHC to apply force to TCR complexes on the T cell surface. Displacement of the bead reveals force-dependent binding and unbinding kinetics. BFP (**d**); BFP uses RBC (red blood cell) as a force transducer (force-sensitive spring) to apply calibrated force between a pMHC-coated bead and TCRs on a cell or bead. Retraction enables real-time measurement of bond lifetimes under force. Flow chamber assay (**e**); shear laminar flow applies tangential force to the cell, which is balanced by the tension force on TCR–pMHC bonds between a cell and surface-coated ligands and the normal force from the surface supporting the cell. Bond lifetimes and rolling behavior under defined hydrodynamic force can be measured. **f**,**g** DNA-based MTP (**f**); a fluorophore-quencher-labeled DNA hairpin unfolds when the T cell exerts force on TCR–pMHC bonds above a threshold, generating fluorescence detectable by TIRF microscopy in a digital manner (on and off). Peptide-based molecular tension sensor (MTS) (**g**); a peptide-based spring linker undergoes force-induced extension, resulting in changes in FRET efficiency for analog quantification of TCR-imposed tension. **h** Force–extension curves of DNA- and peptide-based sensors. Comparison with Myosin-II force (~3 pN per molecule, ~5 nm per stroke) illustrates how sensor choice may affect force sensitivity and extension range.

A point of confusion is whether, and if so, how catch versus slip bonds can be related to binding affinity. The association on-rate, dissociation off-rate and their ratio, that is, binding affinity, of a biomolecular interaction are typically measured by SPR^204^ (Fig. 3a), which assays soluble ligands in fluid phase interacting with receptor ectodomains immobilized on sensor chips. It is referred to as 3D assay because the binding affinity has a unit of 3D volume (that is, reciprocal molar concentration). By contrast, catch and slip bonds refer to different responses of the dissociation off-rate—or, reciprocally, the bond lifetime—to applied force (Fig. 2d). Unlike SPR, where soluble molecules bind to surface-coated receptors in the absence of force, catch and slip bonds must be determined using receptors and ligands coated on opposing surfaces (or cells) to allow implementation of a calibrated force transducer, which also better mimics cell-to-cell or cell-to-extracellular matrix interaction than SPR. Because the dimension of the gap bridged by the interacting molecules between the two surfaces (of an order of 10 nm) is three orders of magnitude smaller than the dimension of a cell (of an order of 10 μm), the cross-junctional molecular interaction is modeled as two-dimensional (2D) binding and affinity is expressed in unit of 2D area (that is, reciprocal surface density)^205–207^ (Fig. 3b). Protein–protein interactions in 3D are typically limited by transport rather than reaction (that is, docking of the two complementary binding sites). Because TCR–pMHC pairs of the same class have the same diffusion coefficient in solution, their 3D on-rates are comparable. As such, their 3D affinity differences mainly reflect differences in off-rate, explaining the desire to relate affinity and bond lifetime. However, this is not the case in 2D, where on-rates exhibit much greater differences^201^, indicating that interactions between molecules anchored on opposing surfaces may be limited by transport rather than docking. Despite theoretical efforts to relate the 2D and 3D affinities^208^, their experimental correlations show mixed results^209–211^, even when comparing the dissociation off-rates, which have the same unit of reciprocal time in both 2D and 3D^201,212–214^. However, catch and slip bonds represent different trends of how the off-rate changes with increasing force and, as such, cannot be determined by the off-rate value at zero force or at any level of force. To this end, a conceptually inconsistent attempt was made to extrapolate results measured by force spectroscopy techniques down to zero force using the Bell equation—a slip-bond model—to match the reciprocal off-rates measured by SPR^50,191,215^, therefore preventing catch bonds from being detected. Meanwhile, force–lifetime profiles only contain information regarding bond dissociation. A simple way to overcome this limitation is to divide the 2D on-rate by the force-dependent off-rate to formulate an effective force-dependent 2D affinity, which includes both association and dissociation information^207,216^.

### Do immunoreceptors exhibit catch bond under force?

A point under debate regarding the mechanosensor model is whether immunoreceptors exhibit distinct responses to mechanical tension applied through bound ligand to exhibit catch versus slip bonds. Historically, the theoretical concept that mechanical force can stabilize receptor–ligand interactions, not just destabilize them, was once debated because it is counterintuitive^165,208,217^. Later, single-molecule (SM) force-clamp experiments demonstrated the existence of catch bonds, where bond lifetimes increase under mechanical tension within a certain range, before decreasing at higher forces^218^. This biphasic force–lifetime pattern stands in contrast to the commonly observed slip bonds, where increasing force monotonically accelerates dissociation (Fig. 2d). Catch bonds have now been observed in a growing number of immunoreceptors^165^. In addition to TCR^42,104,158,166,169,172,198,202,219^, FcγRIIA formed a catch bond with IgG1 Fc^220^, FcγRIII with the nanobody C28^221^, PD-1 forms catch bonds with PD-L1 and PD-L2^222^, CD40 forms catch bond with CD40L^216^, CD28 forms catch bonds with B7-1 and B7-2 (A. M. Rosado and C. Zhu, unpublished data) and BCRs form catch and slip bonds with NIP (4-hydroxy-3-iodo-5-nitrophenylacetic acid) antigen in an Ig isotype-dependent manner (S.T., H.-K.C. and C.Z., unpublished data). These findings suggest a possible link between mechanoregulation of immunoreceptor–ligand dissociation and immunoreceptor-mediated mechanotransduction, and indicate that catch and slip bond behaviors may be of general importance to immunoreceptor mechanosignaling^165^.

In the context of T cell antigen recognition, the idea that TCR forms different types of bonds under tension adds a new dimension to the KPR framework, changing the task of distinguishing two averaged values (dwell times at zero force) to that of distinguishing two averaged curves (bond lifetime versus force of either catch-slip or slip-only type) that contain much more information, thereby greatly reducing the challenge encountered by the antigen discrimination models^42,158,166,167,169,202,219^. Mechanistically, catch bonds may arise from force-induced conformational changes in the receptor–ligand complex. For TCR–pMHC interactions, MD (and steered MD) simulations suggest that mechanical loading can reorient or extend flexible regions within either the TCR or pMHC, stabilizing networks of noncovalent atomic contacts and delaying dissociation^158,166,167,172,173^. In this framework, force does not simply change the differential binding energies of the bond (that is, ligand affinity), but can also dynamically alter the shape of the energy landscape.

Experimental evidence for TCR–pMHC (with and without coreceptor coligation) catch bonds has been most robustly demonstrated using BFP and optical tweezer (OT) force-clamp experiments^42,158,166,167,169,172,202,219^ (Fig. 3c,d). In a landmark study, it was observed that agonist pMHCs formed catch-slip bonds with TCR where lifetimes peaked around 8–15 pN, whereas weaker pMHCs exhibited slip-only bonds^104^. Multiple laboratories have since published bond profiles for 66 TCR–pMHC pairs using either BFP or OT, showing TCR- and pMHC-specific catch and slip bond types^104,158,162,166–168,170,172,198,202,219^. Furthermore, the force–lifetime curve is responsive to mutational manipulations that result in concurrent and correlated changes in both the ability of the mutant TCR–pMHC pairs to form catch bonds and their ability to mediate T cell signaling and function. Moreover, the measured catch versus slip bond properties can be related to submolecular structural changes (for example, interdomain tilting, hinge rotation and stretch)^158,167,223^, which correlate with structural features such as conformational flexibility^162,166,173^. This collection of data suggests that the TCR catch bond is neither idiosyncratic nor restricted to a single system.

Despite compelling evidence, the existence of TCR catch bonds has been challenged by studies using laminar flow chambers (Fig. 3e) in acellular systems using aglycosylated TCRαβ ectodomain proteins coated on beads, which failed to detect catch bonds^215^—even though some of the TCR–pMHC pairs tested by these authors^224^ (for example, 1G4–NY-ESO_157–165_:HLA-A2) were observed to exhibit catch bonds in another laboratory using BFP and cell-surface TCR^167^. These discrepant results underscore the importance of experimental context, which may include several factors. First, the force regimes explored by different techniques vary noticeably. The sensitive BFP and OT are more amenable to low-force measurements, which typically generate at least three points of average lifetime data in the catch regime to ensure that the ascending trend with increasing force is reliable. However, this may be difficult to achieve by flow chambers, possibly due to the technical challenges of decreasing the flow rate to a sufficiently low level. Indeed, the three studies that failed to observed catch bonds measured no more than two data points at and below 10 pN (refs. ^215,224,225^) where bond lifetime peaks in BFP experiments^167^, thus requiring the detection of catch bond to rely on the measurement of a single point in the low-force regime. Because most of the lifetimes were measured at high forces and displayed slip bond trends, the authors fit the Bell equation to the data, making the single data point at the catch bond regime appear as an outlier^215^. Second, an argument for the use of purified proteins is that intrinsic binding properties ought to be measured using cell-free systems to be free of confounding effects from the cell, such as activation. Whereas it is true that repeated cycles of pMHC binding to and pulling on TCR would induce T cell signaling as indicated by intracellular calcium mobilization^104,164^, analysis of lifetimes measured during early versus later phases of consecutive binding–pulling cycles revealed no differences, suggesting that T cell signaling induced in this manner does not alter TCR–pMHC binding properties on the timescale of the experiment (hours)^226^. Meanwhile, it has been shown that force-dependent bond lifetimes evaluated using aglycosylated TCRαβ ectodomains are suppressed compared with those measured in situ where the glycosylated full-length TCRαβ anchored to the T cell membrane through the TM and cytoplasmic domain is associated with CD3s^214^. Variations in force-dependent bond lifetimes were also observed in OT experiments between SM measurements using purified TCRαβ ectodomains and SM–single-cell measurements using the cell-surface full TCR–CD3 complex, further suggesting that the in situ environment enhances the mechanosensory capability of the TCR^166,172^. Moreover, it has been recently shown that mutations in residues at the TCR constant domain (Cβ) in contact with the CD3s impact not only T cell function but also TCR–pMHC catch bonds such that some mutations enhance while other mutations suppress or even completely eliminate catch bond in a fashion that correlates with T cell IL-2 secretion^158^, suggesting allosteric regulation of the catch bond of TCR–pMHC *trans*-interaction by TCRαβ–CD3 *cis*-interaction, which was missing when purified TCRαβ ectodomain proteins were tested for catch bond formation with pMHC. Still, some argue that TCR signaling does not require catch bonds per se, and that any correlation between catch bond profiles and antigen potency may be incidental rather than causal^227^. Others note that, while catch bonds may contribute to signal amplification, they are not necessary for ligand discrimination (or even impair ligand discrimination^215^), which can occur through purely biochemical or spatial mechanisms (for example, KPR or aggregation models). Reconciling these discrepancies requires systematic and careful studies using TCRs of the same sequence, prepared in different ways and measured with different techniques.

### Do immune cells exert endogenous forces on immunoreceptors?

A challenge to the relevance of catch bonds is that it is unclear whether immunoreceptors experience forces under physiological conditions. It has been argued that, for immunoreceptor–ligand bonds across an intercellular junction, even minute cell-level perturbations can generate substantial mechanical loads at the level of individual bonds, particularly under low bond occupancy. For instance, a cell attached to the stationary surface sheared by external fluid flow may experience dislodging forces that must be resisted by adhesion bonds. Migrating cells engage their receptors as traction anchors to generate forward motion or to resist mechanical drag. Even in the absence of bulk cell movement, the retrograde flow of actin filaments beneath the plasma membrane can exert centripetal pulling forces on immunoreceptors bound to immobilized ligands. Indeed, in vitro studies using TFM^228,229^, micropillar array detectors^18,230^, and DNA-based molecular tension probes (MTPs) (Fig. 3f) and tension gauge tethers^18,21,165,222,231–234^ have demonstrated that many cell types (including T and B cells) generate piconewton-scale endogenous, motor-driven and cytoskeleton-dependent pulling forces through immunoreceptor–ligand bonds (including TCR and BCR interacting with pMHC and antigen, respectively) in a signaling-dependent manner^70,231,234,235^ in vitro, although whether and how these results relate to in vivo situations require further study.

It seems reasonable to suggest that the endogenous force the cell exerts on a receptor may relate to the lifetime versus force profile with its ligand because, for the force to be detected, the receptor has to form a durable bond with the ligand to sustain the force. Indeed, existing data suggest an approximate match between the force levels measured by DNA-based MTPs and the force levels at which the catch bonds transition to slip bonds for TCR–pMHC (10–20 pN)^231^, CD40–CD40L (~15 pN)^216^ and BCR–antigen (~20 pN) (S.T., H.-K.C. and C.Z., unpublished data) interactions.

As mentioned previously, lifetime versus force profiles are measured by applying precisely controlled forces on immunoreceptor–ligand bonds (Fig. 3c–e). Interestingly, several receptor–ligand pairs, such as P-selectin–PSGL-1^236^, actin–myosin, integrin α_IIb_β_3_–fibrinogen^237^ and OT1 TCR interacting with OVA pMHC^237^, have been observed to exhibit both catch-slip bonds (when the applied force is held constant, that is, force-clamp) and slip-only bonds (when the applied force is increased linearly in time, that is, force-ramp) using atomic force microscopy, OT and BFP. These represent special cases of a phenomenon known as force–history dependency^236,238–241^. It has recently been shown that cells apply forces on integrins at a rate of ~1 pN/s (refs. ^242–244^). However, it is unknown whether this loading rate is universal or depends on the cell type and receptor. It is also unknown what force waveforms cells exert on their surface receptors and whether, and how, different force waveforms impact receptor–ligand catch bonds.

Yet, not all studies support the presence of such forces, in particular, those that used Förster resonance energy transfer (FRET)-based sensors using spider silk-derived peptides as a strain gauge (Fig. 3g), have reported much smaller forces for integrin (α_5_β_1_ or α_V_β_3_)–ligand (RGD peptide or fibronectin type III 9–10) pair^245,246^ and TCR–pMHC as well as TCR–anti-TCR antibody pairs^247^. For the TCR, Göhring et al. observed 5–8 pN forces using sensor-tagged anti-TCR antibody, but the force level was reduced to ~2 pN when sensor-tagged pMHC was used^247^. The authors suggest that the lower force on the weaker TCR–pMHC interaction could be explained by their short-lived slip bonds. In contrast to these values measured using glass-supported lipid bilayers (SLBs) in a gel-phase, only barely measurable forces below 2 pN were detected when the pMHC-tagged force sensors were anchored to SLBs in the fluid phase^247^.

There may be several explanations for these apparent discrepancies. First, peptide-based FRET sensors are continuous, analog systems that increase nearly linearly with low forces and return to their relaxed state when force is removed^248^ (Fig. 3h). Consequently, they are exquisitely sensitive to small, steady forces but relatively poor at detecting short-lived, impulsive forces, which are characteristic of actomyosin-driven synaptic mechanics. By contrast, DNA-based MTPs operate in a digital manner and do not report signals when small forces (~2 pN), which fall below the lowest force threshold of the MTP used (4.7 pN), are applied. As such, experimenters may tag DNA-based MTPs with ligands at high density to increase signal strength, which emphasizes the detection of higher forces. However, small forces are probably far more prevalent than large ones during cellular interactions. To capture such weak forces at the single-bond level, experimenters may tag peptide-based MTPs with ligands at low density, allowing the detection of low-force events. These distinct detection strategies and mechanical sensitivities probably contribute to the divergent observations made using the two systems. Furthermore, DNA-based probes exhibit digital switching behavior: once the DNA hairpin is unzipped, the rezipping transition upon force removal is not instantaneous. To amplify signal, some experiments were performed in the presence of a locker strand in solution complementary to the unzipped hairpin, allowing transient events to be captured and integrated over time^235^. Moreover, spider silk-based MTPs require short displacements (~5 nm) to reach relatively high force (~15 pN), whereas DNA hairpins take much longer extensions (~15 nm) before appreciable forces (~3 pN) to develop^248,249^ (Fig. 3h). Given the ~5 nm per-stroke characteristic step size of myosin II and a force output of ~3 pN per molecule^250,251^, achieving a high-force output of ~12 pN would require the synchronized action of four to five myosin II motors in a single step. By contrast, for DNA-based MTPs, a single myosin II molecule moving two to three steps could generate sufficient displacement to recruit more than one additional motor, thereby integrating their forces to overcome the resistance required to unfold DNA hairpins with force thresholds of 4.7 or 12 pN. It should be noted that Göhring et al. used antigen-experienced T cell blasts expressing the 5c.c7 TCR to interact with MCC (moth cytochrome c 88-103):IE^k^, a class II pMHC molecule^247^, the force–lifetime profile of which has not been measured to determine the bond type, especially under the loading rate of ~1.5 pN/s measured by these authors. By comparison, Liu et al.^231^ and Ma et al.^233^ used naive T cells expressing OT1 TCR to interact with OVA:H2-K^b^, a class I pMHC molecule that forms a catch-slip bond with lifetime peaks at ~10 pN (ref. ^104^). Interestingly, Ma et al. found no difference in the TCR forces pulling on pMHC anchored at SLBs in either the gel or fluid phases^233^.

Another challenge to the importance of force on the TCR argues that many receptor–ligand pairs are present in the IS, such as LFA-1–ICAM-1 and CD2–LFA-3. These adhesion molecules may bear most of the forces between the T cell and the APC, therefore shielding (or relaxing) the force on TCR–pMHC bonds^232^. Indeed, LFA-1 (integrin α_L_β_2_) is known to bear substantial mechanical loads and forms tight cytoskeletal connections via talin and vinculin^137,252–254^. However, a dual-probe experiment suggests mechanical coupling between LFA-1–ICAM-1 bonds and TCR–pMHC bonds, such that applying forces above 12 pN on LFA-1 increases TCR–pMHC binding and tension, as well as potentiating TCR signaling^232^.

## Perspectives and outlook

The TCR complex is one of the most sophisticated molecular machines evolved to fulfill unique functional requirements: sensitivity, specificity, discrimination, speed, dependency and commonality^39–58^.

Despite substantial progresses from decades of intense research, the inner workings of the TCR that enable these features remain elusive. Two crucial questions persist: (1) How is the information encoded in the antigen peptide received upon pMHC ligation with the TCRαβ at its membrane-distal ligand binding site, transmitted through the receptor structure across the membrane to the cytoplasmic tails of the noncovalently associated CD3s, and transduced into biochemical signals that recode the information as patterns of temporal order, spatial organization and activity levels of signaling molecules^55,88–90^? (2) How are different information contents encoded in different pMHC ligands discriminated to produce distinct T cell outcomes? Our Review suggests that framing the first question as the ‘TCR signal initiation problem’ does not fully account for its complexity. Antigen stimulation does not simply activate the TCR as an on/off switch; rather, this intricate nanomachine interprets information to facilitate decision-making. The second question, usually referred to as the ‘self versus nonself discrimination problem’ also involves more intricacies than its naming would suggest. The TCR’s unique feature of dependency manifests diverse T-lineage cell outcomes. For a given T cell clone expressing an identical TCR sequence, altered peptide ligands can produce not only quantitatively different levels and timings of effector functions but also qualitatively distinct differentiation states and cell fates. These tasks require the TCR to serve as a critical signaling network node, capable not only of delivering antigen-derived information but also of integrating information from other receptors. Moreover, the TCR probably participates in the signaling network’s decision-making process, which depends on the state (for example, naive or activated) of the cell.

Given the above list of requirements, which is probably incomplete, current TCR triggering and discrimination models appear inadequate, notwithstanding that each model probably captures some aspects appropriately. To this end, a few suggested extensions to the existing models seem to be in order. First, signal initiation should be context dependent; for example, it has long been observed that, for the same TCR clone, T cells of different subsets and/or at different differentiation states have different levels of sensitivity to the same antigen. We suggest that triggering and discrimination models should incorporate mechanisms capturing the TCR’s role in this phenomenon. This occurs despite the current paradigm attributing different T cell sensitivities to signals received by other receptors and transduced by intracellular signaling molecules that are differentially expressed across T cells, which, of course, are important. An inference from the context dependency of signal initiation is that the TCR may be capable of inside-out signaling in addition to its well-known role of outside-in signaling^159^, an idea that has already received some experimental support^168^. If inside-out signaling is permitted, it is plausible that the TCR triggering and discrimination machinery could be regulated by the cell to adjust TCR sensitivity, specificity and discriminative power in accordance with the cellular context (for example, by modulating membrane cholesterol levels to regulate the TCR conformational states).

Next, the hypothesis of a regulatable TCR triggering and discrimination machinery offers an explanation for how the T cell may handle the speed versus accuracy trade-off without compromising either, which requires the T cell to recognize foreign peptides at high speed yet still maintain high specificity. A two-phase search strategy, inferred from the regulatable TCR machinery, may allow both requirements to be met. In the first phase, performed at high speed and low accuracy, T cells quickly survey the surface of APCs. Upon positive signal detection, the T cell switches to the second phase, performed at low speed and high accuracy.

A corollary from the above line of reasoning is that triggering and discrimination models should incorporate the serial engagement idea to allow accumulation and integration of intermittent and weak TCR–pMHC interactions, each of which is able to induce brief, transient and reversible signaling intermediates that by themselves may be insufficient to fully active the T cell. Moreover, the serially engaged TCRs may temporarily cooperate. Indeed, using an adhesion frequency assay^206^, it has been observed that a previous TCR engagement can increase the likelihood of future pMHC binding to the same or a nearby TCR, a phenomenon termed molecular memory and quantified by memory index^255^. Specifically, the memory index ($\Delta p$) is the increased probability of having a positive outcome (that is, TCR–pMHC binding) in the current T cell–APC contact under the condition that the immediate past contact yields a positive outcome, relative to the same probability but under a different condition that the immediate past contact yields a negative outcome (that is, no TCR–pMHC binding)^255^. More recently, the above analysis of short-term memory (seconds) has been extended to include memories in intermediate (minutes) and long (hours) timescales^256^. Importantly, the short-term memory has been shown to present only with cell-surface TCR but not purified TCR ectodomain proteins and affect only the on-rate but not the off-rate^226^. Interestingly, this memory effect is inversely correlated with the pMHC potency, can be suppressed by Lck inhibition and is abolished by treatment with choledochal sulfate^256^, but remains unaffected by coreceptor engagement (S.T., H.-K.C. and C.Z., unpublished data). Not only do these recent findings support the temporal cooperativity of the TCR, but they also call for modification of the KPR mode. Specifically, the KPR model should relax the requirement for continuous antigen engagement, remove the assumption that premature disengagement always resets the T cell to its original state, allow not only different antigens but also varying accumulated amounts and durations of signals triggered by the same antigen to produce distinct outcomes, and incorporate feedback mechanisms to account for molecular memory.

The above discussions further reveal the limitations of the conventional approach to examine the TCR discrimination problem by correlating TCR–pMHC interaction characteristics and T cell responses: T cell responses are results not only of stimulations from antigen, but also of inputs from other costimulatory, co-inhibitory, adhesion and accessory molecules, all of which are integrated and processed by the intracellular signaling network that depends on the state of the T cell. Given the potential presence of TCR inside-out signaling, any causative relationship between a single initial input from the TCR and the final decision from the T cell’s signaling network may easily be masked by regulatory mechanisms with feedback controls. New approaches may be required to concurrently measure multiple TCR–pMHC interaction characteristics and observe their immediate consequences. Concomitant measurements are helpful because the further upstream an outcome is observed relative to TCR triggering, the less complex and intertwined the relationship between cause and effect becomes. Multiplexing measurements help increase the information contents and decrease the uncertainty of the cause–effect relationship.

The mechanobiological perspective introduces the possibility that immune recognition involves not only biochemical but also mechanical interactions^17,257,258^. Importantly, mechanotransduction probably integrates well with conformational and allosteric change models: applied forces may propagate through the TCR–pMHC complex to induce structural rearrangements in TCRαβ and CD3 subunits, stabilize the putative active TCR conformation or even facilitate the exposure of ITAMs for phosphorylation^158,162,166,167,173^. However, this mechanical framework faces ongoing controversies, partly due to the heterogeneous mechanical landscape of IS itself. Force is unevenly distributed: peripheral TCRs probably experience higher tension than central TCR clusters. Force profiles can also differ temporally as T cells can apply pulsed, ramped or clutch-like forces. Importantly, T cell activation state plays a crucial role, as naive, effector and exhausted T cells differ in their actomyosin contractility, receptor density and responsiveness to stiffness, which may alter their ability to exert or interpret force^257,259^. Moreover, methodological differences have generated conflicting results regarding the existence and relevance of the TCR catch bond, which is a key aspect of the mechanosenor model.

Despite technical limitations and interpretive challenges, a growing consensus is emerging: T cells are capable of exerting biologically meaningful forces on immunoreceptors, and these forces are both ligand dependent and signaling relevant^258^. However, the field remains divided over the magnitude and necessity of forces^157,162^. Bridging these differences will require more than new measurements; it will demand modeling, biophysical readouts multiplexed with functional assays, and side-by-side comparisons of different force sensors within the same experimental contexts.

The mechanotransduction paradigm provides a rich framework for understanding the adaptive immune system’s exceptional ability to distinguish foreign from self^17,257^. Although the TCR is unique in its clonotypic specificity and pMHC restriction, its mechanosensing capabilities are relevant to other immunoreceptors, making it a general model for studying and solving the immunoreceptor signal initiation problem^87^. The idea of TCR as a platform through which T cells probe the physical nature of antigens is no longer speculative but remains incompletely understood. Despite the need for further development, several tools have already generated a growing body of biophysical and functional data, yet the field has not reached a unified interpretation of these findings. Together, these models support the view that ligand discrimination is governed not only by binding affinity, but also by spatial organization, temporally integrated bond lifetimes and mechanical modulation of binding kinetics, ensuring the six features required for adaptive immune recognition. Thus, the coming decades will be increasingly exciting as more studies are conducted toward a complete understanding of adaptive immune recognition.
